# Public RNA-seq data-based identification and functional analyses reveal that MXRA5 retains proliferative and migratory abilities of dental pulp stem cells

**DOI:** 10.1038/s41598-023-42684-z

**Published:** 2023-09-20

**Authors:** Kazuma Yoshida, Shigeki Suzuki, Hang Yuan, Akiko Sato, Shizu Hirata-Tsuchiya, Masahiro Saito, Satoru Yamada, Hideki Shiba

**Affiliations:** 1 Department of Biological Endodontics, Graduate School of Biomedical and Health Sciences, Hiroshima University, Hiroshima, 734-8553 Japan; 2 Department of Periodontology and Endodontology, Tohoku University Graduate School of Dentistry, Sendai, 980-8575 Japan; 3 Department of Restorative Dentistry, Tohoku University Graduate School of Dentistry, Sendai, 980-8575 Japan

**Keywords:** Dental pulp, Adult stem cells

## Abstract

Dental pulp stem cells (DPSC) usually remain quiescent in the dental pulp tissue; however, once the dental pulp tissue is injured, DPSCs potently proliferate and migrate into the injury microenvironment and contribute to immuno-modulation and tissue repair. However, the key molecules that physiologically support the potent proliferation and migration of DPSCs have not been revealed. In this study, we searched publicly available transcriptome raw data sets, which contain comparable (i.e., equivalently cultured) DPSC and mesenchymal stem cell data. Three data sets were extracted from the Gene Expression Omnibus database and then processed and analyzed. *MXRA5* was identified as the predominant DPSC-enriched gene associated with the extracellular matrix. MXRA5 is detected in human dental pulp tissues. Loss of MXRA5 drastically decreases the proliferation and migration of DSPCs, concomitantly with reduced expression of the genes associated with the cell cycle and microtubules. In addition to the known full-length isoform of MXRA5, a novel splice variant of MXRA5 was cloned in DPSCs. Recombinant MXRA5 coded by the novel splice variant potently induced the haptotaxis migration of DPSCs, which was inhibited by microtubule inhibitors. Collectively, MXRA5 is a key extracellular matrix protein in dental pulp tissue for maintaining the proliferation and migration of DPSCs.

Dental pulp stem cells (DPSC) are isolated from permanent teeth by general mesenchymal stem cell (MSC) markers such as CD44, CD73, CD90, and CD105^1^. Approximately 12% of isolated cells from human dental pulp tissue are identified as DPSCs by single-cell RNA-seq^2,3^. For dental regenerative therapies, DPSCs can differentiate into odontoblasts^4^, and several experimental animal models have shown the usefulness of DPSCs for dental pulp regeneration therapies, especially if combined with various types of growth factors and scaffolds^5^. The peripheral nerve system in the dental pulp tissue, particularly the area adjacent to the odontoblast layer, is well-developed and sensitive to stimuli such as bacteria, thermal changes, and the flow rate of liquid in dentinal tubules. Therefore, DPSCs possess a greater ability to differentiate into neuronal cells, and they have been experimentally applied to regenerate injured nerve tissue and support recovery from paralysis^6–8^. Furthermore, various animal models have been used to demonstrate the therapeutic potential of DPSCs for extraoral cell transplantation therapies in the treatment of skin wound injuries, liver fibrosis, myocardial infarction, and cerebral ischemia^9–14^. The main reason to use DPSCs in extraoral cell regeneration therapies is that teeth are often extracted for clinical purposes, such as non-occlusal third molars, supernumerary teeth, and orthodontic extraction, and the dental pulp tissue is easily accessed if the pulp chamber is opened by dental drilling.

In addition to the application of DPSCs for cell transplantation therapies, endogenous DPSCs may have pivotal roles in maintaining dental pulp tissue homeostasis, similar to MSCs in other tissues^15^. DPSCs usually remain quiescent in dental pulp tissue; however, once the dental pulp tissue is injured and the odontoblast layer is destroyed, DPSCs quickly proliferate and migrate into the injury site, exhibiting immuno-modulatory functions and differentiating into odontoblasts to restore the dentin/pulp complex.

Compared with MSCs, DPSCs possess higher proliferative abilities^16,17^. Similar to other MSCs, FGF-2 and PDGF-BB are pivotal stimulators of DPSC proliferation in vitro^18,19^. However, the key molecules in DPSCs that contribute to greater proliferative and migratory phenotypes have not been revealed. Thus, we performed a whole transcriptome profile analysis by comparing DPSCs with a suitable control, namely MSCs, to identify key molecules and signal pathways, as well as determine the specificity and uniqueness of DPSCs in terms of high proliferation and migration.

In this study, by searching the Gene Expression Omnibus (GEO) database, three whole transcriptome data sets were found in which DPSCs and MSCs were cultured using equivalent methods but with distinct culture systems in each data set, which enabled characterization of the DPSC population in a cross-sectional way with less technical bias. *MXRA5* was identified as the predominant DPSC-up-regulated gene among the genes coding the extracellular matrix, and the roles of MXRA5 in the proliferation and migration of DPSCs were investigated. Moreover, the roles of the newly-identified dental pulp-specific isoform of MXRA5 and the usefulness of its recombinant proteins for DPSC migration were also examined.

## Results

### MXRA5 is selectively expressed by DPSC compared with MSC

**Figure 1:**
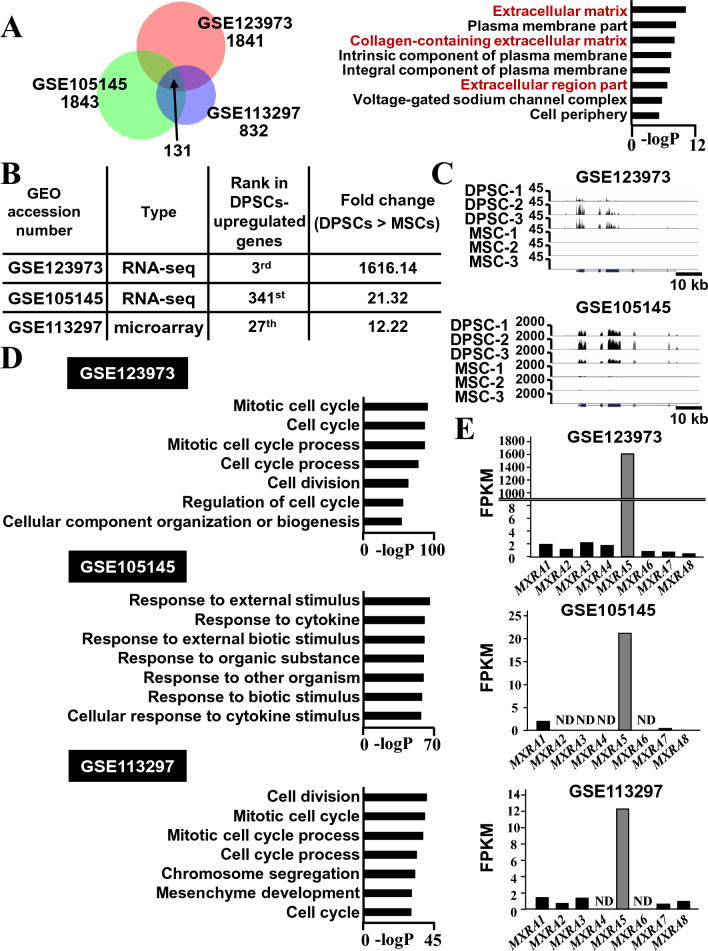
MXRA5 is selectively expressed by extracellular matrix-coding genes in DPSCs. (**A**) Venn diagram of genes selectively expressed in DPSCs compared with MSCs reveals 131 overlapping genes from the three GEO data sets (GSE123973, GSE105145, GSE113297), and the top 8 pathways of DPSC-up-regulated genes from the 131 genes are indicated. (**B**) The type of each GEO data set and the status of MXRA5 in each data set. (**C**) UCSC Genomic Browser tracks of MXRA5 gene locus in GEO data sets (GSE123973, GSE105145). Each data set contains three DPSC and three MSC RNA-seq data points. (**D**) Top 7 pathways of DPSC-up-regulated genes in GSE123973 (1841 genes), GSE105145 (1843 genes), and GSE113297 (832 genes). (**E**) Comparative FPKM ratio of MXRA family genes (MXRA1 to MXRA8) obtained by dividing the FPKM value of DPSCs by those of MSCs. ND = Not detected.

First, to identify the genes selectively expressed by DPSCs compared with MSCs, the GEO database was searched using “dental pulp stem cells” and “human,” and “RNA-seq.” As a result, 15 data sets were extracted, and among these, two RNA-seq data sets (GSE123973^20^ and GSE105145^21^) contained the raw data of RNA-seq analyses for the DPSCs and MSCs. Furthermore, the GEO database was searched using “dental pulp stem cells” and “human,” and “microarray.” As a result, 29 data sets were extracted, and among these, a microarray data set (GSE113297^22^) contained the raw data of RNA-seq analyses for the DPSCs and MSCs. Thus, the raw data of GSE123973, GSE105145, and GSE113297 were downloaded. Then, the gene expression profiles were analyzed using bioinformatics tools, as described in the “Methods” section. GSE123973, GSE105145, and GSE113297 contained 1841, 1843, and 832 differentially expressed genes that possessed more than twofold higher expression levels in DPSCs compared with MSCs, respectively. Among these identified genes, 131 genes were commonly up-regulated in DPSCs among the three data sets (Fig. 1A). The Gene Ontology (GO) analysis of the cellular component of the 131 genes showed enrichment for genes related to the extracellular environment such as the “extracellular matrix,” “collagen-containing extracellular matrix,” and “extracellular region part.” Among the 131 commonly up-regulated genes, *MXRA5* was top-ranked in the genes coding extracellular proteins. *MXRA5* was ranked 3rd for the DPSC-up-regulated genes in the GSE123973 data set (Fig. 1B). *MXRA5* was ranked 341st among the 1843 and 27th among the 832 DPSC-up-regulated genes in the GSE105145 and GSE113297 data sets, respectively. UCSC Genomic Browser analysis of the two RNA-seq data sets revealed DPSC-specific tag accumulation in the *MXRA5* gene locus (Fig. 1C). GO analyses of the biological processes for the genes up-regulated in DPSCs compared with MSCs in the GSE123973 and GSE113297 data sets revealed the enrichment of genes associated with cell cycle such as “mitotic cell cycle,” “cell cycle,” “mitotic cell cycle process,” “cell cycle process,” and “cell division.” These results imply that DPSCs possess strong proliferative properties compared with MSCs in these experiments (Fig. 1D). This cell cycle-associated term enrichment was more profound in the GSE123973 data set, in which *MXRA5* expression was also the most highly selective compared with GSE105145 and GSE113297. MXRA5 belongs to the MXRA family consisting of MXRA1, MXRA2, MXRA3, MXRA4, MXRA5, MXRA6, MXRA7, and MXRA8. The FPKM expression ratio between DPSCs and MSCs of all *MXRAs* revealed that *MXRA5* is the most selective DPSC-specific gene in the GSE123973, GSE105145, and GSE113297 data sets (Fig. 1E).

### MXRA5 is expressed in dental pulp tissue in vivo and selectively expressed in DPSCs

**Figure 2:**
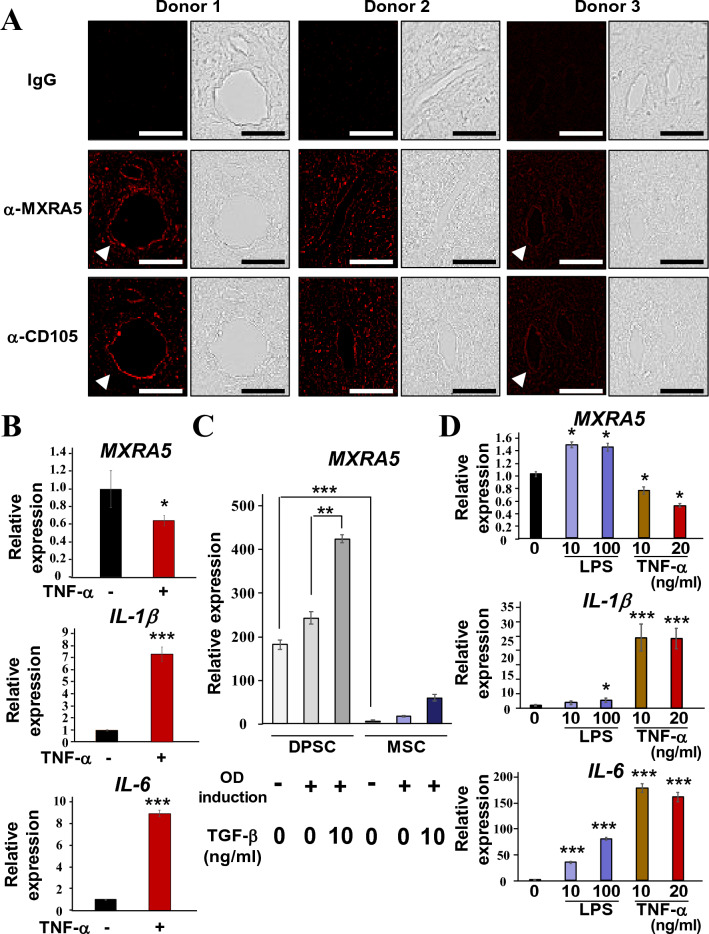
MXRA5 detection in vivo for dental pulp tissue and physiologically strong expression of MXRA5 in DPSCs. (**A**) Expression of MXRA5 and CD105, a marker of stem cells, in normal dental pulp tissues extracted from orthodontic extracted teeth. Fixed dental pulp tissues were stained with α-MXRA5 and α-CD105. Perivascular tissue was enlarged. (**B**) Extracted dental pulp tissues were divided in half and each half was either stimulated with or without TNF-α for 24 h, and then the total RNA was extracted to analyze *MXRA5*, *IL-1β*, and *IL-6* expression. (**C**) DPSCs and MSCs were simultaneously cultured in the presence or absence of TGF-β1 under odontogenic induction by supplementation with ascorbic acid (50 μg/ml) and β-glycerophosphate (10 mM) for 3 days, and the total RNA was collected to analyze *MXRA5*. (**D**) DPSCs were stimulated with LPS (0, 10, and 100 ng/ml) or TNF-α (0, 10, and 20 ng/ml) for 24 h, and the total RNA was collected to analyze *MXRA5*, *IL-1β*, and *IL-6* expression. Each column represents the mean ± SD, where n = 4 for each group in (**B**, **D**) and n = 3 for each group in (**C**). Scale bars correspond to 50 μm. *p < 0.05; ***p < 0.001 significantly different from the non-treated cells (**B**, **D**).

To examine the in vivo MXRA5 expression in human dental pulp tissue, whole pulp tissues were extracted from 3 premolars from different donors, and fixed pulp tissues were stained with MXRA5 and CD105, a marker of stem cells. MXRA5 and CD105 were detected in entire pulp tissue particularly in the perivascular tissue (arrow heads, Fig. 2A). Then, dental pulp tissues were newly extracted from premolars and organ-cultured for 24 h in the presence of TNF-α (10 ng/ml), and *MXRA5* expression was significantly decreased (Fig. 2B). Increased *IL-1β* and *IL-6* expression by TNF-α validated the inflammatory response of extracted dental pulp tissues against TNF-α. To validate the predominant expression pattern of *MXRA5* in DPSCs compared with MSCs in the three GEO data sets, DPSCs and MSCs were equivalently cultured in our laboratory, and as expected, *MXRA5* was 24-fold more highly expressed in our DPSCs than MSCs (Fig. 2C). Note that TGF-β1 strongly induced *MXRA5* expression when DPSCs were cultured for 3-days in odontogenic induction medium. Then, DPSCs were cultured in the presence of LPS (10 and 100 ng/ml) or TNF-α (10 and 20 ng/ml), and the *MXRA5* expression level was increased by LPS and decreased by TNF-α (Fig. 2D). Increased *IL-1β* and *IL-6* expression by LPS and TNF-α validated the inflammatory response of DPSCs against LPS and TNF-α. These results suggest that MXRA5 was specifically expressed by DPSCs.

### MXRA5 suppression decreases the expression of genes associated with the cell cycle

To delineate the roles of MXRA5 in DPSCs, two independent specific siRNAs for *MXRA5* were generated. si-*MXRA5*-1 and si-*MXRA5*-2 targeted exon 7 and the 3′UTR region, respectively (Table 1).

**Table 1:** Target sequences for siRNA duplex.

Oligo name	Sense strand (5′ → 3′)	Antisense strand (5′ → 3′)	Location
si*-MXRA5*-1	UUAUGAUACUGCUUGUUUGCU	CAAACAAGCAGUAUCAUAACC	ex7 of full length MXRA5 (= ex2 of PV-MXRA5)
si*-MXRA5*-2	UGAAUGUUCCUCAGAUAUCCU	GAUAUCUGAGGAACAUUCAUC	3′UTR
si-control	GUACCGCACGUCAUUCGUAUC	UACGAAUGACGUGCGGUACGU	–

**Figure 3:**
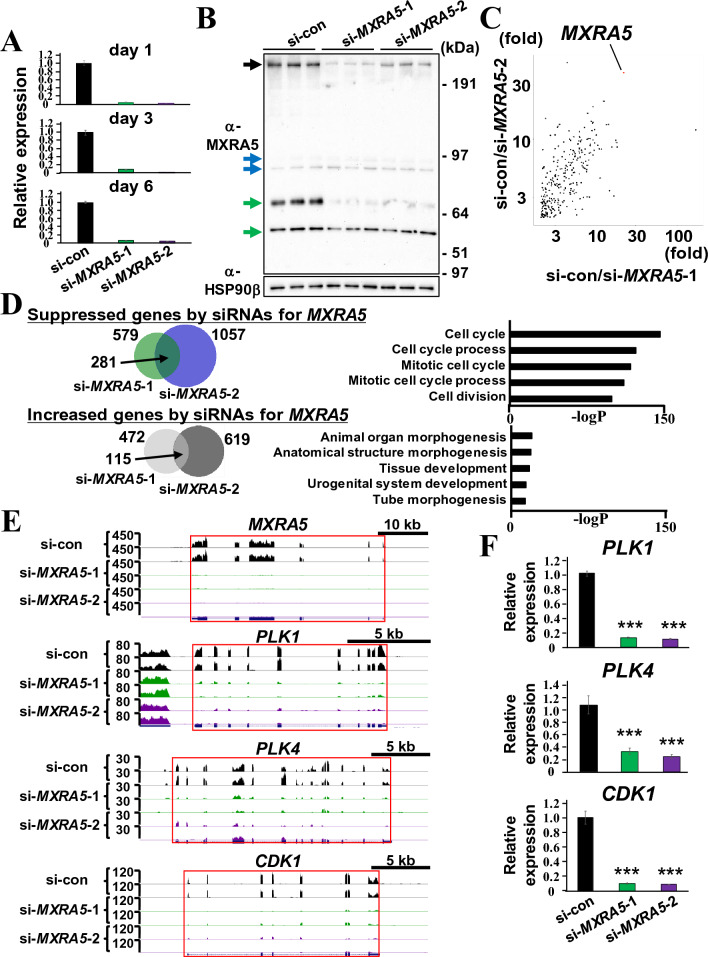
Suppression of MXRA5 expression inhibits cell cycle-related gene expression in DPSCs. (**A**, **B**) DPSCs were transfected with si-control, si-*MXRA5*-1, or si-*MXRA5*-2. *MXRA5* mRNA expression (**A**) and MXRA5 secretion (**B**) were analyzed using *HPRT* for normalization and extracellular HSP90β for loading amount validation. The black arrow indicates the MXRA5-specific band. Blue and green arrows indicate non-specific bands. Original blots are presented in Supplementary Fig. 3. (**C**) Total RNAs were collected from DPSCs transfected with si-control, si-*MXRA5*-1, or si-*MXRA5*-2, and whole-genomic transcriptional changes were assessed by RNA-seq. The scatter plot shows a comparison of the FPKM ratio obtained by dividing the FPKM value of si-con-DPSCs by that of si-*MXRA5*-1-DPSCs (x-axis) and by dividing the FPKM value of si-con-DPSCs by that of si-*MXRA5*-2-DPSCs (y-axis). (**D**) The upper Venn diagram of genes down-regulated by si-*MXRA5*-1 transfection (579 genes) and genes down-regulated by si-*MXRA5*-2 transfection (1057 genes) reveals 281 overlapping genes. The lower Venn diagram of genes up-regulated by si-*MXRA5*-1 transfection (472 genes) and genes up-regulated by si-*MXRA5*-2 transfection (619 genes) reveals 115 overlapping genes. The top 5 pathways of commonly down-regulated and up-regulated genes from the 281 and 115 overlapping genes are indicated, respectively. (**E**) UCSC Genomic Browser tracks of *MXRA5*, *PLK1*, *PLK4*, and *CDK1* genes show exon-specific tag accumulation in si-con-DPSC duplicates and a drastic decrease in tag accumulation in si-*MXRA5*-1-DPSC and si-*MXRA5*-2-DPSC duplicates. (**F**) Suppressed expression of *PLK1*, *PLK4*, and *CDK1* confirmed by qPCR. Each column represents the mean ± SD, and n = 3 for each group in (**A**, **F**). ***p < 0.001 significantly lower than si-con-DPSC.

Then DPSCs were transfected with these two siRNAs and successful reduction of *MXRA5* was observed at days 1, 3, and 6 (Fig. 3A). SDS-PAGE analysis of the concentrated supernatant of DPSCs transfected with either control siRNA (DPSC-si-con), si-*MXRA5*-1 (DPSC-si-*MXRA5*-1), or si-*MXRA5*-2 (DPSC-si-*MXRA5*-2) showed reduced MXRA5 in the supernatant of DPSC-si-*MXRA5*-1 and DPSC-si-*MXRA5*-2 compared with that of DPSC-si-con (black arrow, Fig. 3B). Non-specific bands are indicated by blue and green arrows. Equivalent amounts of concentrated supernatant loaded into each lane were validated by the amounts of extracellular HSP90β. Then, the fold change of each expressed gene between DPSC-si-*MXRA5*-1 and DPSC-si-con was calculated to divide the FPKM value of DPSC-si-con by that of DPSC-si-*MXRA5*-1 (si-con/si-*MXRA5*-1). Similarly, the fold change of each expressed gene between DPSC-si-*MXRA5*-2 and DPSC-si-con (si-con/si-*MXRA5*-2) was calculated. Then, the si-con/si-*MXRA5*-1 and si-con/si-*MXRA5*-2 ratios of each gene were dot-blotted (Fig. 3C). Even though a few genes were strongly suppressed by either si-*MXRA5*-1 or si-*MXRA5*-2, none of the genes, except *MXRA5*, were clearly suppressed by both si-*MXRA5*-1 and si-*MXRA5*-2. This result suggests that non-specific gene expression reduction by si-*MXRA5*-1 and si-*MXRA5*-2 and common phenotypic features in DPSC-si-*MXRA5*-1 and DPSC-si-*MXRA5*-2 should arise from the suppression of MXRA5.

Fold change analyses between DPSC-si-con and DPSC-si-*MXRA5*-1 and between DPSC-si-con and DPSC-si-*MXRA5*-2 revealed that 579 and 1057 transcripts were suppressed by siRNAs for *MXRA5*, respectively. Among these transcripts, 281 genes, including *MXRA5*, were commonly suppressed (Fig. 3D). GO analysis of the biological process of the 281 genes revealed that cell cycle-related terms, such as “cell cycle,” “cell cycle process,” “mitotic cell cycle,” “mitotic cell cycle process,” and “cell division,” were enriched. The entire list of enriched terms (− logP <  − 40) is provided in Supplemental Fig. 1. In contrast, between DPSC-si-con and DPSC-si-*MXRA5*-1 and between DPSC-si-con and DPSC-si-*MXRA5*-2, 472 and 619 transcripts were up-regulated by siRNAs for *MXRA5*, respectively. Among these transcripts, 115 genes were commonly up-regulated. GO analysis of the biological process of the 115 genes revealed that some of the terms, such as “animal organ morphogenesis,” “anatomical structure morphogenesis,” “tissue development,” “urogenital system development,” and “tube morphogenesis,” were enriched. However, their p-values were relatively high compared with those of commonly suppressed genes. UCSC Genome Browser analysis of *MXRA5* and three representative cell cycle-related genes, namely *PLK1*, *PLK4*, and *CDK1,* revealed tag enrichments in the exon region of these gene loci of DPSC-si-con, but nominal tags were found in that of DPSC-si-*MXRA5*-1 and DPSC-si-*MXRA5*-2 (Fig. 3E). Concomitantly, suppressed expression of *PLK1*, *PLK4*, and *CDK1* in DPSC-si-*MXRA5*-1 and DPSC-si-*MXRA5*-2 was confirmed by qPCR analyses (Fig. 3F).

### MXRA5 suppression inhibits the proliferation and migration of DPSCs

**Figure 4:**
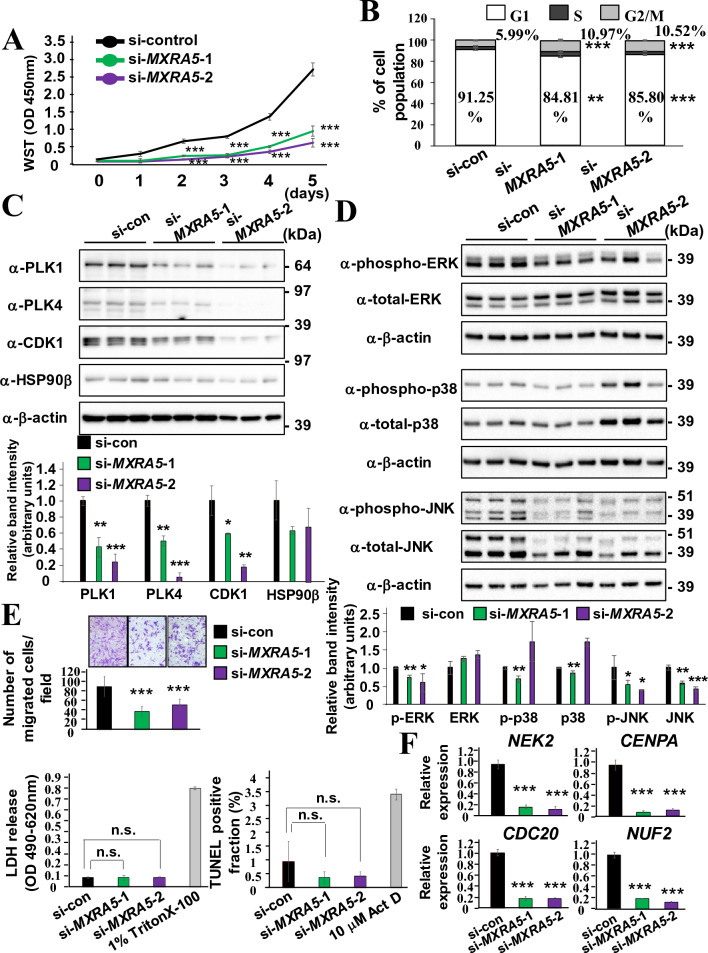
Suppression of MXRA5 expression inhibits cell proliferation and migration in DPSCs. (**A**–**F**) DPSCs were transfected with si-control, si-*MXRA5*-1, or si-*MXRA5*-2. Knockdown of MXRA5 inhibited cell proliferation (**A**), increased cell population in G2/M phases (**B**), inhibited PLK1, PLK4, and CDK1 expression (**C**), inhibited phosphorylation of ERK and JNK, and total JNK expression (**D**), inhibited migration toward 10% serum (**E**), and suppressed microtubule-related genes (**F**). The LDH release and TUNEL-positive fraction of siRNA-transfected cells were examined, and the treatment with 1% Triton X-100 and Actinomycin D (Act D) was conducted as an experimental positive control (**E**). Original blots are presented in Supplementary Figs. 4, 5, 6, and 7. Each column represents the mean ± SD, where n = 5 for each group in (**A**), n = 4 for each group in (**E**), and n = 3 for each group in (**B, C, D, F**). *p < 0.05; **p < 0.01; ***p < 0.001 significantly lower than DPSCs transfected with si-control at each time point (**A**), higher than DPSCs transfected with si-control (**B**), lower than DPSCs transfected with si-control (**C, D, E, F**).

Next, to clarify the outcome of reduced expression in cell cycle-related genes in DPSC-si-*MXRA5*-1 and DPSC-si-*MXRA5*-2, an equal number of DPSC-si-con, DPSC-si-*MXRA5*-1, and DPSC-si-*MXRA5*-2 were seeded onto 96-well plates, and the increase in cell number was comparatively evaluated by WST assay (Fig. 4A). DPSC-si-*MXRA5*-1 and DPSC-si-*MXRA5*-2 showed significantly reduced cell proliferation from day 2 to day 5 compared with DPSC-si-con. When DPSC-si-con, DPSC-si-*MXRA5*-1, and DPSC-si-*MXRA5*-2 cells were re-activated with serum-containing medium for 24 h, after performing 24 h-serum starvation, the cell cycle was stuck in the G2/M phase in DPSC-si-*MXRA5*-1 and DPSC-si-*MXRA5*-2 cells (Fig. 4B). Then, the transcribed products of cell cycle-related genes, including PLK1, PLK4, and CDK1, in DPSC-si-*MXRA5*-1 and DPSC-si-*MXRA5*-2 were quantified by SDS-PAGE analyses and compared with DPSC-si-con using β-actin for normalization (Fig. 4C). PLK1, PLK4, and CDK1 were significantly suppressed in DPSC-si-*MXRA5*-1 and DPSC-si-*MXRA5*-2. In contrast, the expression level of HSP90β, a non-cell cycle-related protein, was not significantly altered.

To investigate the effects of MXRA5 knockdown in DPSCs for the activation of mitogen-activated protein kinase (MAPK), the phosphorylation degree and total amount of ERK, p38, and JNK were evaluated by SDS-PAGE analyses (Fig. 4D). Phosphorylation of ERK and JNK, but not p38, was significantly decreased in DPSC-si-*MXRA5*-1 and DPSC-si-*MXRA5*-2 compared with DPSC-si-con (Fig. 4D). Additionally, the total amount of JNK, but not ERK or p-38, was significantly decreased in DPSC-*MXRA5*-1 and DPSC-si-*MXRA5*-2. These results indicate that the ERK signal activity was modulated by phosphorylation status, and the JNK signal activity was modulated by the decrease of JNK expression and concomitant phosphorylation status when *MXRA5* expression was suppressed. Neither phosphorylation nor expression of p-38 was altered by *MXRA5* knockdown. Next, the roles of MXRA5 in chemotaxis cell migration from the serum-free upper chamber to the serum-containing lower chamber were evaluated by Boyden chamber assay. DPSC-si-*MXRA5*-1 and DPSC-si-*MXRA5-*2 exhibited significantly decreased numbers of migrated cells compared with DPSC-si-con (Fig. 4E). LDH and terminal deoxynucleotidyl transferase (TdT)-mediated dUTP-biotin nick end labeling (TUNEL) assays of DPSCs transfected with siRNAs indicated that transfection of si-*MXRA5*-1 and si-*MXRA5-*2 did not differentially induce cell death or cell apoptosis compared with transfection of si-con. DPSCs were treated with 1% of Triton X-100 for the LDH assays and 10 μM of Actinomycin D for TUNEL assays, used as positive control experiments. These results verified that the decrease in cell migration was specifically attributed to the down-regulation of MXRA5 and not due to cell apoptosis or cell death. As shown in Supplemental Fig. 1, which lists the original top-ranked terms of the common si-*MXRA5*-down-regulated genes (Fig. 3D), in addition to cell cycle-related terms (colored blue), 3 microtubule-related pathways (colored red) were ranked. The expression changes in *NEK2*, *CENPA*, *CDC20*, and *NUF2*, which were classified into microtubule-related terms by the GO analyses and are known to support microtubule-related cell migration, were comparatively analyzed, and all these genes were down-regulated in DPSC-si-*MXRA5*-1 and DPSC-si-*MXRA5-*2 compared with DPSC-si-con (Fig. 4F).

### Novel splicing isoforms of MXRA5 promote DPSC proliferation and migration

**Figure 5:**
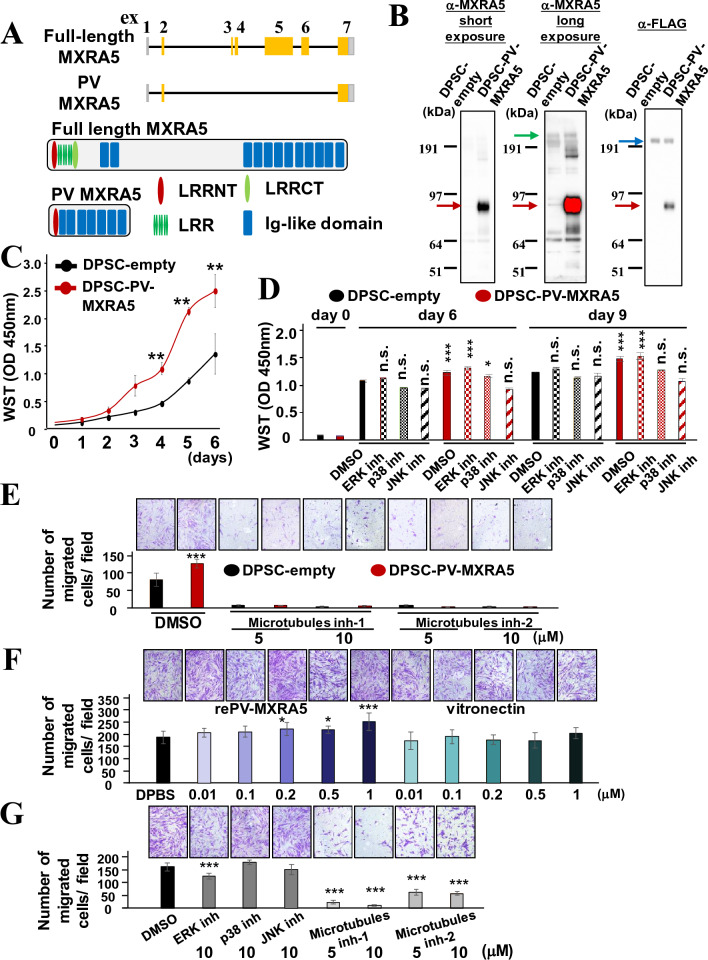
Novel splicing variant of MXRA5 enhances cell proliferation and migration in DPSCs. (**A**) Schematic exon views and protein functional motifs of two isoforms of MXRA5. (**B**) Stable transfection of pulp variant MXRA5 (PV-MXRA5) into DPSC to generate DPSC-PV-MXRA5 was validated by SDS-PAGE analyses. The red arrow indicates exogenous PV-MXRA5. The green arrow indicates full-length MXRA5. The blue arrow indicates a non-specific band. (**C**) DPSC-PV-MXRA5 showed aggressive proliferation compared with DPSC transfected with the control plasmid (DPSC-empty). Original blots are presented in Supplementary Fig. 8. (**D**) MAPK inhibitors differentially decreased accelerated proliferative properties of DPSC-PV-MXRA5. (**E**) Transwell plates were seeded with DPSC-empty or DPSC-PV-MXRA5 in the upper chamber in the presence or absence of microtubules inhibitors, paclitaxel and combretastatin A4, and cell migration toward 10% serum was evaluated. (**F**) Undersides of the transwell membranes were pre-coated with the indicated concentrations of rePV-MXRA5 and vitronectin, seeded with DPSC in serum-free medium, and incubated for 16 h. (**G**) Undersides of the transwell membranes were pre-coated with 0.2 M rePV-MXRA5. DPSCs were seeded with microtubule inhibitors at the indicated concentrations and incubated for 16 h. After washing non-adherent cells, the attached cells were stained with 0.2% crystal violet and dissolved in 1% SDS solution. Absorbance was measured at 570 nm. Each column represents the mean ± SD, where n = 5 for each group in (**C**, **D**) and n = 4 for each group in (**E**, **F**, **G**). *p < 0.05; **p < 0.01; ***p < 0.001 significantly higher than DPSC-empty (**C**) and higher than DMSO-treated DPSC-empty at each time point (**D**), higher than DMSO-treated DPSC-empty (**E**), higher than DPSC seeded onto DPBS-coated chamber (**F**), and lower than DMSO-treated DPSC (**G**). n.s. means not significantly higher than DMSO-treated DPSC-empty, which indicates that a higher number of cells obtained by PV-MXRA5 was eliminated by the addition of MAPK inhibitors.

By conducting PCR using the primer pairs associated with the 2nd and last exons, two bands were visualized (Supplemental Fig. 2). Sequence reads of amplified products revealed two isoforms of *MXRA5* that were expressed by DPSCs. The larger one is the known full-length MXRA5 and the smaller one is the novel MXRA5 isoform lacking exons 3, 4, 5, and 6 (Fig. 5A). From the schematic views shown in Fig. 5A, the amino acid sequence indicated that the novel splicing isoform, namely the pulp variant of MXRA5 (PV-MXRA5), lacked the leucine-rich repeat N-terminal (LRRNT) cap, leucine-rich repeat (LRR), and the leucine-rich repeat C-terminal (LRRCT) cap, but retained most of the immunoglobulin-like (Ig-like) domains. Entire amino acid sequences of full-length MXRA5 and PV-MXRA5 are shown in Supplemental Fig. 2. To examine the effects of full-length MXRA5 and PV-MXRA5 overexpression for the proliferation of DPSCs, DPSCs stably expressing either full-length MXRA5 or PV-MXRA5 tagged with FLAG were generated. However, because of the large size of full-length MXRA5 (2828 amino acids), we were unable to establish DPSCs stably expressing substantial levels of exogenous full-length MXRA5. Consequently, only DPSCs overexpressing PV-MXRA5 (DPSC-PV-MXRA5) were used for the gain of function assays. Expression of transgenes tagged with FLAG was identified using anti-MXRA5 and anti-FLAG antibodies (Fig. 5B). Next, DPSC-PV-MXRA5 and DPSCs stably expressing empty expression plasmids (DPSC-empty) were cultured for 6 days, and the numbers of cells in 96-well plates were counted by WST assay (Fig. 5C). DPSC-PV-MXRA5 showed a significantly higher number of cells on day 4 compared with DPSC-empty. Treatment with MAPK inhibitors showed that SP600125, a JNK inhibitor, significantly decreased the cell numbers of DPSC-PV-MXRA5 toward that of DPSC-empty at day 6 and day 9, and SB203580, a p38 inhibitor, significantly decreased the cell numbers of DPSC-PV-MXRA5 toward that of DPSC-empty at day 9. FR180204, an ERK1/2 inhibitor, did not decrease the cell numbers of DPSC-PV-MXRA5 (Fig. 5D).

Next, DPSC-empty and DPSC-PV-MXRA5 were seeded onto the upper chamber of the Boyden chamber, and chemotaxis cell migration was evaluated (Fig. 5E). DPSC-PVMXRA5 showed a significantly higher number of migrated cells. The addition of microtubule inhibitor-1 (paclitaxel) and microtubule inhibitor-2 (combretastatin A4) drastically inhibited cell migration (Fig. 5E).

Next, to evaluate the effect of PV-MXRA5 protein on DPSC migration, recombinant PV-MXRA5 (rePV-MXRA5) was generated using a mammalian expression system, and haptotaxis migration toward rePV-MXRA5 was investigated. The lower surface of the Boyden chamber was coated with various concentrations of either recombinant PV MXRA5 or vitronectin (0, 0.01, 0.1, 0.2, 0.5, and 1.0 μM), and an equal number of DPSCs was seeded onto the upper chamber. Vitronectin was comparatively evaluated because it is a well-known adhesion and migration substrate for many types of cells. The number of migrated cells was significantly increased if rePV-MXRA5 was seeded at 0.2, 0.5, and 1.0 μM (Fig. 5F). However, the number of migrated cells toward vitronectin was not significantly altered compared with DPBS-coated (equivalent to non-coated). Finally, the effects of microtubule inhibitors on haptotaxis cell migration of DPSCs toward rePV-MXRA5 (0.2 μM) were investigated (Fig. 5G). The number of DPSCs that migrated toward rePV-MXRA5 was drastically inhibited in the presence of microtubule inhibitors (5 and 10 μM).

## Discussion

By analyzing public data sets containing DPSC and MSC transcriptome raw data, the present study demonstrated that MXRA5 is predominantly expressed by DPSCs, and MXRA5 is required for maintaining the strong proliferative and migratory properties of DPSCs by inducing cell cycle- and microtubule-related gene expression. Furthermore, recombinant proteins of the novel MXRA5 variant can potently induce haptotaxis migration of DPSCs.

Since three terms were associated with the extracellular matrix among the top 8 GO terms for common DPSC-enriched genes in all GEO data sets (Fig. 1A), the potent extracellular matrix secretion of DPSCs may be based on the potent regeneration ability of DPSCs. For example, the combination of polylactic acid with a decellularized extracellular matrix scaffold obtained from a DPSC culture enhanced the bone regeneration of experimental calvarial bone defects compared with polylactic acid alone^23^. The experimental details of the cell culture procedures used in GSE123973, GSE105145, and GSE113297, such as seeding density, passage number, serum concentration, and serum source, were different. However, it should be noted that *MXRA5* was often highly ranked as a DPSC-up-regulated gene (Fig. 1B), showed a higher DPSC/MSC fold change (Fig. 1B, C), and possessed the highest fold change among the MXRA family of genes (Fig. 1E). TNF-α stimulation, which suppresses DPSC proliferation and migration^24^, down-regulated *MXRA5* in DPSCs (Fig. 2) in vitro and in vivo. To examine the effects of TNF-α for *MXRA5* expression in dental pulp tissues, we conducted organ culturing, which retained the cell-to-cell and ECM-to-cell communications. IHC indicated that not only DPSCs, which is around 12% of human dental pulp tissue^2,3^, but also other dental pulp tissue residential cells may express a certain amount of *MXRA5*. Notably, the reduced expression of *MXRA5* shown in Fig. 2C was caused by dental pulp residential cells, rather than DPSCs alone. Notably, cell cycle-related terms, such as “mitotic cell cycle,” “cell cycle,” “mitotic cell cycle process,” and “cell cycle process,” were predominantly enriched in GSE123973, in which the highest ranking of *MXRA5* was observed compared with GSE105145 and GSE113297 (Fig. 1D). These trends indicate the positive role of MXRA5 in cell proliferation. As expected, it was revealed that cell cycle-related terms were selectively enriched in suppressed pathway terms by MXRA5 knockdown (Fig. 3) and drastically decreased in cell proliferation by MXRA5 knockdown (Fig. 4).

Proteins belonging to the Polo-like kinase (Plk) family, such as PLK1, PLK2, and PLK4, are serine/threonine kinases that regulate the cell cycle, particularly the control of sister chromatid separation^25^. Therefore, the loss of PLK1 or pharmacological inhibition of PLK activity resulted in slower cell proliferation concomitantly with spindle checkpoint blockade and G2/M phase arrest in tumor cells^26,27^. Consistently, a drastic decrease of PLK1 and PLK4 induced cell cycle arrest at the G2/M phase in DPSCs (Fig. 4B). MXRA5 suppression in WPMY-1 cells, the SV40 large T antigen immortalized stromal cell line, also inhibited cell proliferation, but in contrast to DPSCs, cell cycle arrest was identified in the G0/1 phase with down-regulation of CDK4, CDK2, Cyclin D1, and Cyclin A1/2^28^. This result indicated that different cell cycle-related target genes are relied on by different cell types. The mechanism by which MXRA5 suppression results in the down-regulation of cell cycle-related genes remains unknown in all cell types. However, in the promoter region of PLK1, there are transcriptional factor consensus DNA sequences for E2A, AP-1, AP2, SP1, and NFκB. Moreover, Forkhead transcription factors were reported to bind to the *PLK1* promoter and induce *PLK1* expression^29,30^. Among the Forkhead transcription factors, FoxM1, which is expressed in a cell cycle-dependent manner, regulated *PLK1* expression. As FoxM1 functions were directly connected with Raf-MEK mediated MAPK activation^31^, MXRA5-induced MAPK activation possibly activates PLK expression by promoting transcriptional activities of Forkhead transcription factors in *PLK* gene loci. MAPKs, including ERK1/2, p38, and JNK pathways, have been identified as key intracellular signaling pathways triggered by MXRA5^28,32,33^. MXRA5 overexpression induced trophoblast cell invasion, and both the p38 and ERK1/2 inhibitors suppressed the MXRA5-induced invasive ability^32,33^. MXRA5 suppression resulted in the decreased phosphorylation of ERK1/2 and p38 without alteration of the total amount of ERK1/2 and p38 in the above trophoblast cells. Thus, similar to trophoblast cells and immortalized stromal cells, the genetic gain and loss of function assays revealed that MXRA5 positively regulated DPSC proliferation and migration (Figs. 4 and 5). However, the downstream pathway of MAPKs stimulated with MXRA5 appears to be different. As shown in Fig. 4, the MXRA5-dependent cell proliferation property of DPSCs relied on the ERK1/2 and JNK signal pathways but not the p38 pathway. Moreover, PV-MXRA5 enhanced cell proliferation depending on p38 and JNK (Fig. 5G). Even though the receptors of MXRA5 remain unknown, the different types, combinations, and interactions of MAPK upstream molecules, such as MAP4Ks and MAP3Ks^34^, are assumed to be involved in the activation of different MAPK downstream pathways among different cell types.

Target sequences of si-*MXRA5*-1 and si-*MXRA5*-2 are common for full-length MXRA5 and PV-MXRA5. However, endogenous PV-MXRA5 expression was undetectable owing to lower expression levels (Figs. 3B and 5B). The estimated core protein size of PV-MXRA5 is 76.41 kDa, as shown in Supplemental Fig. 2. There were two slight bands in the vicinity of exogenous PV-MXRA5 (α-MXRA5 long exposure, Fig. 5B). However, these bands were non-specific because the band intensity was not reduced by the transfection of si-*MXRA5*-1 and si-*MXRA5*-2 (blue arrows, Fig. 3B). Therefore, phenotypic changes obtained by si-*MXRA5*-1 and si-*MXRA5*-2 were consequences of knockdown in both isoforms but mainly relied on full-length MXRA5. Full-length MXRA5 consists of 2828 amino acids, and the estimated core protein size is 312.15 kDa, as shown in Supplemental Fig. 2. Thus, it has been difficult to prepare DPSCs overexpressing full-length MXRA5 and full-length recombinant MXRA5 protein because of the lower transfection efficiency of the expression vector into DPSCs and recombinant protein-expressing cells, the low amount of recombinant protein secretion from the recombinant protein-expressing cells, and insufficient enrichment of recombinant protein during the purification process. Instead, PV-MXRA5 was prepared, and its inducible roles for cell migration were disclosed (Fig. 5). Because vitronectin induced the haptotaxis migration of osteosarcoma MG63 cells and dental pulp fibroblastic cells^35,36^, DPSCs were also seeded onto the vitronectin-coated Boyden chamber. Considering that PV-MXRA5, but not vitronectin, induced haptotaxis migration of DPSCs, vitronectin might not be suitable for inducing DPSC migration, and PV-MXRA5 might be useful for specifically inducing DPSCs if applied as pulp direct capping materials and for accelerating dentin/pulp tissue wound healing. Ig-like domains of MXRA5, which are mostly conserved in PV-MXRA5, might be key for enhancing the migration of DSPCs. Ig-like domains are one of the frequently-identified protein domains recognized in various types of protein families and contribute to various molecular pathways such as ligand recognition by T cell antigen receptors, cell adhesion, and protein–protein binding^37,38^. Thus, Ig-like domains in MXRA5 may act as ligands of unknown receptors or extracellular competitors of ligand binding to receptors containing Ig-like domains for ligand recognition, and consequently, induce the expression of microtubule-related genes. Microtubules are major components in the cytoskeleton of eukaryotic cells and contribute to cell organization, especially for relatively larger cells such as MSCs^39,40^. However, microtubules dynamically polymerize and de-polymerize to modulate their length, and this dynamism is required for cell migration. Thus, up-regulation of microtubule-related genes by MXRA5 may be key for MXRA5-induced DPSC migration, although further studies are necessary to identify the functional amino acid part of MXRA5, MXRA5 receptors, and the intracellular molecular mechanisms linked with the induction of microtubule-related gene expression.

Similar to trophoblasts^32^, TGF-β1 dose-dependently induced MXRA5 expression in DPSCs (Fig. 2B). Therefore, the Smad signal, a main intracellular signaling pathway of TGF-β1, is key for MXRA5 expression. Approximately 700 types of proteins were identified from mineralized dentin, and bioactive proteins, such as TGF-β1, DSP, and DPP, are thought to be secreted from dentin to induce protective reactions, such as reparative dentin formation, when the dentin/pulp complex is invaded by oral bacteria because of severe caries and exposure caused by tooth fracture, severe attrition, or acidic conditions^41–45^. Unexpectedly, LPS induced *MXRA5* expression, unlike TNF-α, which suppressed it, even though LPS and TNF-α both commonly activate the NF-κB pathway. It has been reported that different combinations of TLR ligands/receptors commonly activated NF-κB signaling, but the resultant epigenetic and genetic modifications differ^46^. Thus, we speculate that such a specific regulatory system of *MXRA5* expression by the NF-κB pathway must exist in DPSCs. NF-κB is known to inhibit Smad guiding to Smad-responsive elements and subsequent Smad transcriptional activity^47^. Therefore, the balance among TGF-β1 secreted from dentin, LPS from pathogenic bacteria, and TNF-α from immunological cells and pulp residential cells is key for *MXRA5* expression in DPSCs during dentin/pulp healing processes and reparative dentin formation.

The present results demonstrated for the first time that MXRA5 is one of the key extracellular proteins that differentiate DPSCs from MSCs, and MXRA5 contributes to the proliferative and migratory properties of DPSCs through the induction of Pol family proteins, which are required for cell proliferation, and microtubule-related molecules, which are required for cell migration. Therefore, as a potential therapeutic for clinical use, MXRA5 may have genetic and extracellular applications in producing large-scale cultures of high-quality DPSCs and improving the outcomes of dental pulp wound healing therapies, such as pulp capping, by enhancing DPSC infiltration to the injury site.

## Methods

### Ethics

Dental pulp tissues were collected in compliance with the Hiroshima University ethical guidelines for epidemiological research. All experimental procedures were approved by the Committee of Research Ethics at Hiroshima University (Permit Number: E2021-2741). Informed consent was obtained from all participants of the study.

### Bioinformatics analyses

The GEO database was searched using “DPSC” or “dental pulp stem cells” and “MSC,” and as a result, two RNA-seq data sets (GSE123973^20^ and GSE105145^21^) and a microarray data set (GSE113297^22^) were identified. The raw RNA expression data obtained from MSCs and DPSCs in GSE123973, GSE105145, and GSE113297 were downloaded and extracted. For processing of the RNA-seq data sets (GSE123973, GSE105145), adapter trimming was conducted using Trim Galore version 0.6.6 (http://www.bioinformatics.babraham.ac.uk/projects/trim_galore/) with default settings and then aligned to a reference genome (hg38) using HISAT2 version 2.2.1^48^. Tag directories were generated using “makeTagDirectory” in HOMER^49^, and gene expression at the exons was quantified using the “analyzeRepeast.pl” command in HOMER with “-strand both” and “-count exons” to identify differentially expressed genes using 3 raw data points of independent DPSC lines as targets and three raw data points of independent MSC lines as backgrounds in both the GSE123973 and GSE105145 data sets. For analyzing the microarray data set (GSE113297), expression profiles of all genes (GSE113297.top.table.tsv) were downloaded. Collectively, in the three GSE data sets (GSE123973, GSE105145, and GSE113297), the expression ratio of all transcripts was calculated by dividing the expression level in DPSCs by that in MSCs (DPSC/MSC), and the transcripts with DPSC/MSC ratios of more than 2 were extracted.

For analyzing whole-genomic gene expression changes in DPSCs after *MXRA5* suppression, total RNA was purified from DPSCs transfected with si-*MXRA5*-1, si-*MXRA5*-2, or si-control. Purified RNA was DNase-treated and used in RNA-seq analyses as described previously^50^. Raw data sets were processed as described above, and tag directories were generated using “makeTagDirectory” in HOMER^49^. To identify the differentially expressed genes by the treatment with si-*MXRA*5-1, gene expression at the exons was quantified using the “analyzeRepeast.pl” command in HOMER with “-strand both” and “-count exons” for DPSCs treated with si-control as the target and DPSCs treated with si-*MXRA5*-1 as the background. The expression ratio was calculated for all obtained transcripts by dividing the expression level in DPSCs treated with si-control by that in DPSCs treated with si-*MXRA5*-1 (si-control/si-*MXRA5*-1), and the transcripts with si-control/si-*MXRA5*-1 ratios of more than 2 were extracted as suppressed genes by *MXRA5* suppression and those with ratios of less than 0.5 were extracted as induced genes by *MXRA5* suppression. Similarly, the differentially suppressed genes by the treatment with si-*MXRA5*-2 were identified, and si-control/si-*MXRA5*-2 was calculated. Regarding visualization in the UCSC Genome Browser, BAM files were converted to bigwig files using “bamCoverage” in deeptools with binsize = 10, minMappingQuality 10^51^.

### Cell culture and stable cell generation

Human DPSCs and MSCs were purchased from Lonza Inc. (Walkersville, MD). DPSCs and MSCs were expanded in a specified medium (DPSC: #PT-3005, DPSC BulletKit, Lonza Inc., MSC: #PT-3001, MSCGM BulletKit, Lonza Inc.) and then maintained in low glucose Dulbecco’s Modified Eagle Medium (DMEM; Thermo Fisher Scientific, Carlsbad, CA) supplemented with 100 units/ml of penicillin, 100 μg/ml of streptomycin, and 10% fetal bovine system for at least 2 passages before the experiments. DPSCs and MSCs were cultivated at 37 °C under humidified atmospheric conditions (5% CO_2_ and 95% air). The pulp variant of MXRA5 tagged with FLAG at the C-terminal (PV-MXRA5-FLAG) was amplified from the DPSC cDNA generated using SSIV (Thermo Fisher Scientific) with reverse primers having the FLAG coding sequence. Then, the amplified PV-MXRA5-FLAG was ligated into pLVSIN-CMV-Pur Vector (Takara Bio Inc., Otsu, Japan) to obtain pLVSIN-CMV-Pur-PV-MXRA5-FLAG, and the sequences were verified. Then, DPSCs stably overexpressing PV-MXRA5-FLAG (DPSC-PV-MXRA5) and control (DPSC-empty) cells were generated as described previously^52^.

### Quantitative PCR (qPCR) analysis

Total RNA from DPSCs and MSCs was purified, and cDNA was prepared as described previously^50,53^. Human *HPRT* was used as an internal reference control. PCR primer sequences for target genes are shown in Table 2.

**Table 2:** Primer pairs.

Primer name	Direction	Sequence
*MXRA5*	Forward	GGATGAGGGAGGAAGGAGAC
	Reverse	AAGTCTTGTTCCGGATGGTG
*IL-1β*	Forward	TCCAGGAGAATGACCTGAGC
	Reverse	GTGATCGTACAGGTGCATCG
*IL-6*	Forward	TACATCCTCGACGGCATCTC
	Reverse	TTTCAGCCATCTTTGGAAGG
*PLK1*	Forward	AACACGCCTCATCCTCTACAAT
	Reverse	AGGAGGGTGATCTTCTTCATCA
*PLK4*	Forward	CCACAGACAACAATGCCAAC
	Reverse	GGTCTGCAAATGGAAAAGGA
*CDK1*	Forward	GATTCTATCCCTCCTGGTC
	Reverse	AATATGGTGCCTATACTCC
*NEK2*	Forward	ATGTTTTCCTGGATGGCAAG
	Reverse	TGCGATTCATTTGTTCAGGA
*CENPA*	Forward	TCCGAAAGCTTCAGAAGAGC
	Reverse	AGGCGTCCTCAAAGAGATGA
*CDC20*	Forward	GAGGTGCAGCTATGGGATGT
	Reverse	ACATCATGGTGGTGGATGTG
*NUF2*	Forward	AGTTGACTGCCTGCCTTCAT
	Reverse	TTTGGTCCTCCAAGTTCAGG
*HPRT*	Forward	TGGCGTCGTGATTAGTGATG
	Reverse	CGAGCAAGACGTTCAGTCCT

### Tissue extraction and immunohistochemistry

Healthy teeth were extracted for orthodontic purposes with informed consent, as described previously^54^. Briefly, the pulp chamber was opened by minimum drilling and pulp tissue was removed from the chamber using a barbed broach. For TNF-α stimulation, extracted pulp tissue was stimulated with and without 10 ng/ml of TNF-α (210-TA, R&D Systems, Minneapolis, MN) in DMEM with 10% serum for 24 h, and then, total RNA was extracted. For immunostaining, extracted pulp tissue was fixed using 4% paraformaldehyde in DPBS at 4 °C for 24 h. They were then dehydrated by passing through a graded ethanol series, placed in xylene, and embedded in paraffin. Immunostaining was performed on 5 µm-thick paraffin sections as described previously^52,55^. The primary antibodies used for immunostaining are described in Table 3.

**Table 3:** Antibodies for immunohistochemsitry.

Antibodies for immunohistochemistry		Concentration
α-MXRA5	25,472-1-AP, Proteintech	Final concentration 1 μg/ml
α-CD105	28,117-1-AP, Proteintech	Final concentration 1 μg/ml
rabbit IgG	DA1E, Cell signaling technologies	Final concentration 1 μg/ml

Antibodies for immunodetection		Dilution ratio
α-MXRA5	25,472–1-AP, Proteintech	1:1000
α-PLK1	GTX104302, GeneTex Inc	1:1000
α-PLK4	GTX111754, GeneTex Inc	1:1000
α-CDK1	GTX108120, GeneTex Inc	1:1000
α-HSP90β	GTX09012, GeneTex Inc	1:2000
α-β-actin	GTX109639, GeneTex Inc	1:5000
α-phospho ERK	#4370, Cell signaling technologies	1:1000
α-ERK	#4695, Cell signaling technologies	1:1000
α-phospho p38	#4511, Cell signaling technologies	1:1000
α-p38	#8690, Cell signaling technologies	1:1000
α-phospho JNK	#4668, Cell signaling technologies	1:1000
α-JNK	#9252, Cell signaling technologies	1:1000
α-FLAG	F3165, Sigma-Aldrich	1:1000
HRP-conjugated goat anti-rabbit IgG	#7074: Cell signaling technologies	1:2000
HRP-conjugated goat anti-mouse IgG	SA00001-1, Proteintech	1:2000

Chemical inhibitors		
SB203580	AG-CR1-0030-M005, AdipoGen	
SP600125	1496/10, R&D Systems	
FR180204	F1214, Medchem express LLC	
Paclitaxel	S1150, Selleck chemicals	
Combretastatin A4	S7783, Selleck chemicals	

### Transient transfection of siRNA

RNA sequences for targeting *MXRA5* by siRNA were selected using Enhanced siDirect, a web-based target-specific siRNA design software. Control siRNA was previously described^53,56,57^. siRNAs were generated by Sigma-Aldrich. The siRNA sequences of two types of siRNAs for *MXRA5* (si-*MXRA5*), and that of control siRNA (si-control), are shown in Table 1. siRNAs were forward-transfected into DPSCs at a final concentration of 10 nM using Lipofectamine RNAiMAX reagent (Thermo Fisher Scientific) and then incubated for 24 h.

### Cell proliferation assay

DPSCs were seeded at a density of 1.5 × 10^3^ cells/well onto a 96-well plate and then cultured. The next day of the seeding was set as day 0, and the medium was replaced every 3 days. The number of DPSCs was quantified using the Cell Counting Kit-8 (Dojindo, Kumamoto, Japan).

### Cell cycle determination

Cells were trypsinized and collected, and the cell cycle phase was determined using the cell cycle phase determination kit (Cayman Chemical, ANN Arbor, MI). After the staining procedure, flow cytometry was conducted using a 488 nm excitation laser.

### Immunoblotting

For detecting secreted MXRA5, the supernatant of DPSC transfectants was precipitated and 40-fold concentrated with trichloroacetic acid, and precipitants were washed twice with acetone before reducing them. For detecting intracellular proteins, DPSCs were seeded onto a 24-well plate, and the next day, cells were transfected with siRNAs for 24 h. Then, the medium was replaced with DMEM supplemented with 10% serum for 3 days. Next, DPSCs transfected with control siRNA were directly lysed with 100 μl of LDS sample buffer (Thermo Fisher Scientific) and DTT (Thermo Fisher Scientific) before reducing. Similarly, DPSCs transfected with either si-*MXRA5*-1 or si-*MXRA5*-2 were directly lysed with 50 μl of LDS sample buffer and DTT before reducing. Because the number of cells decreased by MXRA5 knockdown, half of the LDS sample buffer with DTT for DPSCs transfected with control siRNA was used for DPSCs transfected with either si-*MXRA5*-1 or si-*MXRA5*-2. Then, reduced samples were loaded onto NuPAGE Bis–Tris (Thermo Fisher Scientific) gels in MOPS buffer for immunodetection with the primary and secondary antibodies described in Table 3.

### LDH release assay

LDF assay was conducted as described previously^55^. DPSC cells were transfected with siRNAs for 24 h as described above, and then the supernatants were collected. To conduct a positive control experiment for the LDH release assay, DPSCs were treated with 1% Triton X-100 for 24 h, and then the supernatants were collected. The LDH release assay was performed using the LDH Cytotoxicity Detection Kit (Takara Bio Inc.). The supernatants were mixed with an equal amount of the detection buffer containing diaphorase, NAD+, and tetrazolium. The mixtures were incubated for 20 min at room temperature and quantified by measuring absorbance at 490 and 600 nm as reference wavelengths using a microtiter plate reader (Multiskan, Life Technologies).

### TUNEL assay

DPSC cells were transfected with siRNAs for 24 h as described above. As a positive control of apoptotic induction, DPSC cells were treated with 10 μM of Actinomycin D (FUJIFILM Wako Pure Chemical Corporation, Japan) for 48 h. Then, apoptotic DPSC cells were detected by TUNEL assay using Apoptosis Detection Kit (Takara bio, Japan). For quantification, DAPI-positive nuclei associated with siRNAs transfection or Actinomycin D treatment were considered and the fraction of TUNEL-positive nuclei was determined. At least 14,000 siRNA-transfected cells and 5700 Actinomycin D-treated cells were examined.

### Preparation and purification of recombinant MXRA5-pulp variant protein

For detecting full-length and pulp variant MXRA5 in DPSCs, PCR was prepared with forward primers (GACAAGATGCCCAAGCGCGCGCACTGGGGGGCCCTCT) binding to exon 2, which includes nucleotides coding the start codon, and reverse primers (TCAGAAGACGTGGATGTAAGTTGTTTTGGAGTCACTG) binding to exon 7, which includes nucleotides coding the stop codon, and the 2 amplified products were sequenced. PV-MXRA5 cDNA used for generating pLVSIN-CMV-PV-MXRA5 was cloned into the pCEP4-Mul-PURD expression vector^35,58–60^ such that the amino-terminus of recombinant proteins was 6 × His-tagged. The PV-MXRA5 vector was transfected into 293EBNA cells using X-tremeGENE (Roche, Indianapolis, IN) and incubated for 24 h. The transfection medium was replaced with a growth medium containing puromycin (5 μg/ml), and transfected cells were then cultured for 3 days. Surviving cells were routinely cultured with puromycin (0.5 μg/ml) and used in the expression of the His-tagged recombinant PV-MXRA5 (rPV-MXRA5) protein, and rPV-MXRA5 was purified from the supernatants as described previously^35,58–60^.

### Migration assay

Chemotaxis and haptotaxis cell migration were assayed using Transwell migration chambers with a pore size of 8 μm (353,097 cell culture-treated, Falcon System Inc., Columbia, MD). For haptotaxis assays, the undersides of the membranes were coated at room temperature overnight with 100 μl of various concentrations of rePV-MXRA5 proteins or human vitronectin (354,238, BD Biosciences, Bedford, MA), followed by 2 washes with DPBS. Subconfluent DPSCs were rinsed twice with DPBS, harvested using 5 mM EDTA in DPBS for 10 min at room temperature, collected by centrifugation, and then suspended in serum-free DMEM. These cells were seeded onto the upper chamber at a density of 1.5 × 10^5^ cells/well in 150 μl of serum-free DMEM for chemotaxis assay and 10% serum-containing DMEM for haptotaxis migration assays in the presence or absence of chemical inhibitors (Table 3). The lower chamber was filled with 600 μl of 10% serum-containing DMEM. After 16-h incubation at 37 °C, cells that had migrated were stained and counted using 7 randomly selected fields at 200 × magnification, as described previously^35^. The median images were visualized in the figures.

### Statistical analysis

Statistical analysis was performed by one-way analysis of variance, followed by the Bonferroni test (Figs. 2C, D, 3, 4, and 5) and two-tailed unpaired Student’s t-tests (Fig. 2B).

### Supplementary Information

Supplementary Information 1.

Supplementary Information 2.

Supplementary Information 3.

Supplementary Information 4.

Supplementary Information 5.

Supplementary Information 6.

Supplementary Information 7.

Supplementary Information 8.

Supplementary Information 9.

Supplementary Information 10.

Supplementary Information 11.

Supplementary Information 12.

## Data Availability

The original raw data of RNA-seq analysis have been deposited in the NCBI GEO database with accession number GSE228136.
