# Supplementary material for: Public RNA-seq data-based identification and functional analyses reveal that MXRA5 retains proliferative and migratory abilities of dental pulp stem cells
Source: Sci Rep. 2023 Sep 20;13:15574. doi: 10.1038/s41598-023-42684-z (PMC10511426; doi:10.1038/s41598-023-42684-z)
Supplement: Supplementary file 1 — Supplementary Information 1. [file 41598_2023_42684_MOESM1_ESM.pdf]

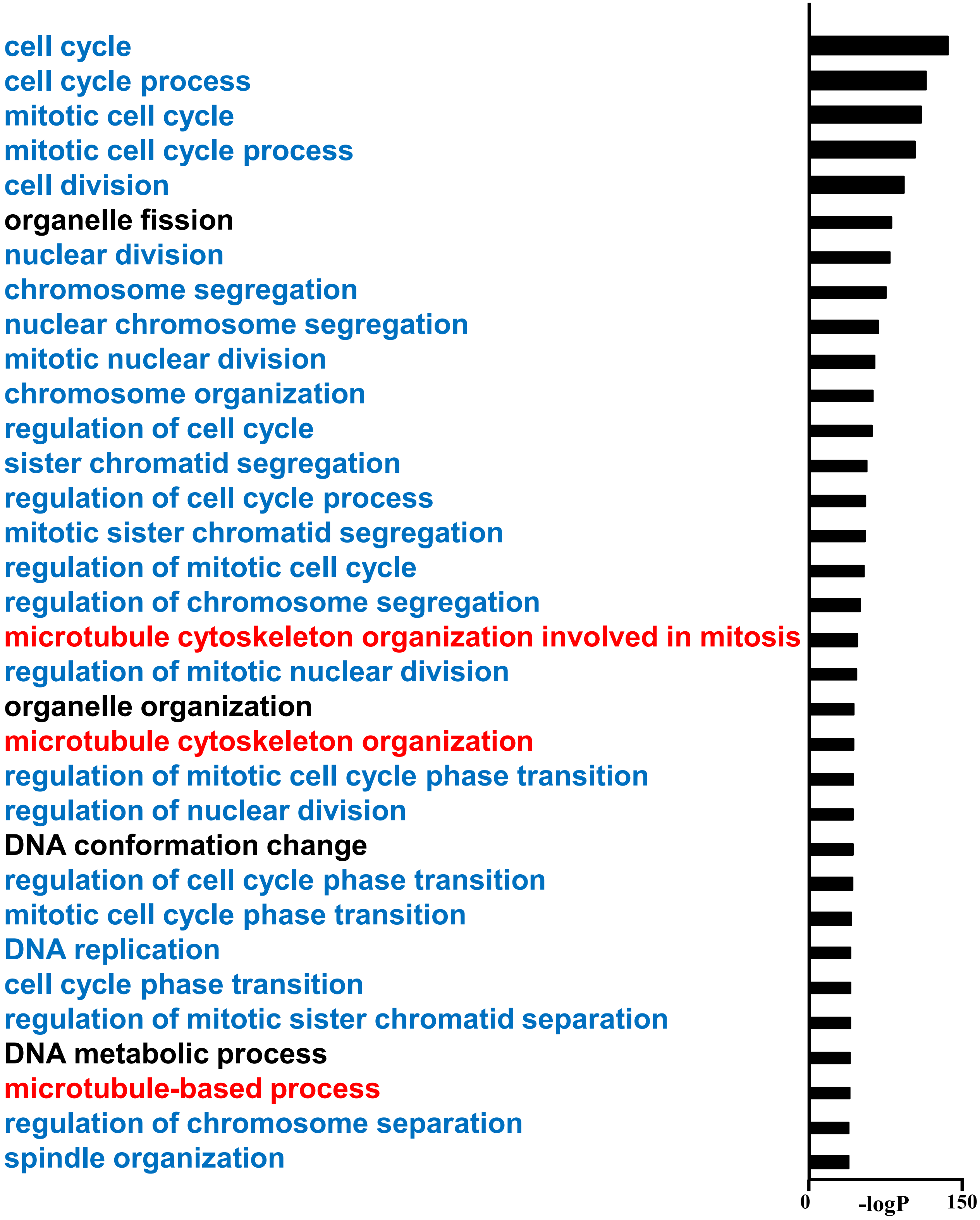


**Supplemental Figure 1. Top-ranked terms enriched as common si-*MXRA5*-down-regulated genes**

**Full length MXRA5**

**2828 AA**

**312.15 kDa**

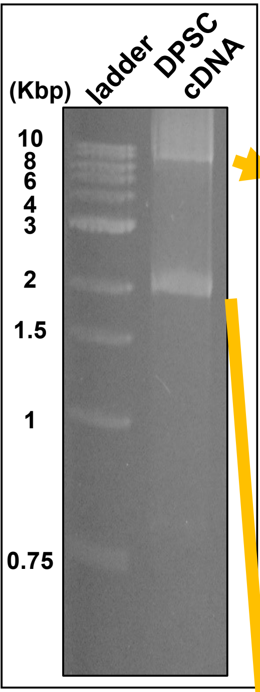


MPKRAHWGALSVVLILLWGHPRVALACPHPCACYVPSEVHCTFRSLASVPAGIAKHVERINLGFNSIQA
LSETSFAGLTKLELLMIHGNEIPSIPDGALRDLSSLQVFKFSYNKLRVITGQTLQGLSNLMRLHIDHNKIE
FIHPQAFNGLTSLRLLHLEGNLLHQLHPSTFSTFTFLDYFRLSTIRHLYLAENMVRTLPASMLRNMPLLE
NLYLQGNPWTCDCEMRWFLEWDAKSRGILKCKKDKAYEGGQLCAMCFSPKKLYKHEIHKLKDMTCLK
PSIESPLRQNRSRSIEEEQEQEEDGGSQLILEKFQLPQWSISLNMTDEHGNMVNLVCDIKKPMDVYKIH
LNQTDPPDIDINATVALDFECPMTRENYEKLWKLIAYYSEVPVKLHRELMLSKDPRVSYQYRQDADEEA
LYYTGVRAQILAEPEWVMQPSIDIQLNRRQSTAKKVLLSYYTQYSQTISTKDTRQARGRSWVMIEPSG
AVQRDQTVLEGGPCQLSCNVKASESPSIFWVLPDGSILKAPMDDPDSKFSILSSGWLRIKSMEPSDSG
LYQCIAQVRDEMDRMVYRVLVQSPSTQPAEKDTVTIGKNPGESVTLPCNALAIPEAHLSWILPNRRIIND
LANTSHVYMLPNGTLSIPKVQVSDSGYYRCVAVNQQGADHFTVGITVTKKGSGLPSKRGRRPGAKAL
SRVREDIVEDEGGSGMGDEENTSRRLLHPKDQEVFLKTKDDAINGDKKAKKGRRKLKLWKHSEKEPE
TNVAEGRRVFESRRRINMANKQINPERWADILAKVRGKNLPKGTEVPPLIKTTSPPSLSLEVTPPFPAIS
PPSASPVQTVTSAEESSADVPLLGEEEHVLGTISSASMGLEHNHNGVILVEPEVTSTPLEEVVDDLSEK
TEEITSTEGDLKGTAAPTLISEPYEPSPTLHTLDTVYEKPTHEETATEGWSAADVGSSPEPTSSEYEPPL
DAVSLAESEPMQYFDPDLETKSQPDEDKMKEDTFAHLTPTPTIWVNDSSTSQLFEDSTIGEPGVPGQS
HLQGLTDNIHLVKSSLSTQDTLLIKKGMKEMSQTLQGGNMLEGDPTHSRSSESEGQESKSITLPDSTL
GIMSSMSPVKKPAETTVGTLLDKDTTTATTTPRQKVAPSSTMSTHPSRRRPNGRRRLRPNKFRHRHK
QTPPTTFAPSETFSTQPTQAPDIKISSQVESSLVPTAWVDNTVNTPKQLEMEKNAEPTSKGTPRRKHG
KRPNKHRYTPSTVSSRASGSKPSPSPENKHRNIVTPSSETILLPRTVSLKTEGPYDSLDYMTTTRKIYS
SYPKVQETLPVTYKPTSDGKEIKDDVATNVDKHKSDILVTGESITNAIPTSRSLVSTMGEFKEESSPVGF
PGTPTWNPSRTAQPGRLQTGIPVTTSGENLTDPPLLKELEDVDFTSEFLSSLTVSTPFHQEEAGSSTTL
SSIKVEVASSQAETTTLDQDHLETTVAILLSETRPQNHTPTAARMKEPASSSPSTILMSLGQTTTTKPAL
PSPRISQASRDSKENVFLNYVGNPETEATPVNNEGTQHMSGPNELSTPSSDQDAFNLSTKLELEKQV
FGSRSLPRGPDSQRQDGRVHASHQLTRVPAKPILPTATVRLPEMSTQSASRYFVTSQSPRHWTNKPEI
TTYPSGALPENKQFTTPRLSSTTIPLPLHMSKPSIPSKFTDRRTDQFNGYSKVFGNNNIPEARNPVGKP
PSPRIPHYSNGRLPFFTNKTLSFPQLGVTRRPQIPTSPAPVMRERKVIPGSYNRIHSHSTFHLDFGPPA
PPLLHTPQTTGSPSTNLQNIPMVSSTQSSISFITSSVQSSGSFHQSSSKFFAGGPPASKFWSLGEKPQI
LTKSPQTVSVTAETDTVFPCEATGKPKPFVTWTKVSTGALMTPNTRIQRFEVLKNGTLVIRKVQVQDRG
QYMCTASNLHGLDRMVVLLSVTVQQPQILASHYQDVTVYLGDTIAMECLAKGTPAPQISWIFPDRRVW
QTVSPVEGRITLHENRTLSIKEASFSDRGVYKCVASNAAGADSLAIRLHVAALPPVIHQEKLENISLPPG
LSIHIHCTAKAAPLPSVRWVLGDGTQIRPSQFLHGNLFVFPNGTLYIRNLAPKDSGRYECVAANLVGSA
RRTVQLNVQRAAANARITGTSPRRTDVRYGGTLKLDCSASGDPWPRILWRLPSKRMIDALFSFDSRIK
VFANGTLVVKSVTDKDAGDYLCVARNKVGDDYVVLKVDVVMKPAKIEHKEENDHKVFYGGDLKVDCV
ATGLPNPEISWSLPDGSLVNSFMQSDDSGGRTKRYVVFNNGTLYFNEVGMREEGDYTCFAENQVGK
DEMRVRVKVVTAPATIRNKTYLAVQVPYGDVVTVACEAKGEPMPKVTWLSPTNKVIPTSSEKYQIYQD
GTLLIQKAQRSDSGNYTCLVRNSAGEDRKTVWIHVNVQPPKINGNPNPITTVREIAAGGSRKLIDCKAE
GIPTPRVLWAFPEGVVLPAPYYGNRITVHGNGSLDIRSLRKSDSVQLVCMARNEGGEARLILQLTVLEP
MEKPIFHDPISEKITAMAGHTISLNCSAAGTPTPSLVWVLPNGTDLQSGQQLQRFYHKADGMLHISGLS
SVDAGAYRCVARNAAGHTERLVSLKVGLKPEANKQYHNLVSIINGETLKLPCTPPGAGQGRFSWTLPN
GMHLEGPQTLGRVSLLDNGTLTVREASVFDRGTYVCRMETEYGPSVTSIPVIVIAYPPRITSEPTPVIYT
RPGNTVKLNCMAMGIPKADITWELPDKSHLKAGVQARLYGNRFLHPQGSLTIQHATQRDAGFYKCMA
KNILGSDSKTTYIHVF

**Pulp variant-MXRA5 (PV-MXRA5)**

**698 AA**

**76.41 kDa**

MPKRAHWGALSVVLILLWGHPRVALACPHPCACYVPSEVHCTFRSLASVPAGIAKHVERINLGFDSRIK
VFANGTLVVKSVTDKDAGDYLCVARNKVGDDYVVLKVDVVMKPAKIEHKEENDHKVFYGGDLKVDCV
ATGLPNPEISWSLPDGSLVNSFMQSDDSGGRTKRYVVFNNGTLYFNEVGMREEGDYTCFAENQVGK
DEMRVRVKVVTAPATIRNKTYLAVQVPYGDVVTVACEAKGEPMPKVTWLSPTNKVIPTSSEKYQIYQD
GTLLIQKAQRSDSGNYTCLVRNSAGEDRKTVWIHVNVQPPKINGNPNPITTVREIAAGGSRKLIDCKAE
GIPTPRVLWAFPEGVVLPAPYYGNRITVHGNGSLDIRSLRKSDSVQLVCMARNEGGEARLILQLTVLEP
MEKPIFHDPISEKITAMAGHTISLNCSAAGTPTPSLVWVLPNGTDLQSGQQLQRFYHKADGMLHISGLS
SVDAGAYRCVARNAAGHTERLVSLKVGLKPEANKQYHNLVSIINGETLKLPCTPPGAGQGRFSWTLPN
GMHLEGPQTLGRVSLLDNGTLTVREASVFDRGTYVCRMETEYGPSVTSIPVIVIAYPPRITSEPTPVIYT
RPGNTVKLNCMAMGIPKADITWELPDKSHLKAGVQARLYGNRFLHPQGSLTIQHATQRDAGFYKCMA
KNILGSDSKTTYIHVF

**Supplemental Figure 2. Amino acid sequence of full-length MXRA5 and novel pulp splicing variant of MXRA5** Identification of two isoforms of *MXRA5* in DPSCs by qPCR expanding from the 2nd exon, including the start codon, to the last exon, including the stop codon (A). The amino acid sequences colored in green are derived from exon 2 of full-length MXRA5 (MXRA5) and exon 2 of novel pulp splicing variant of MXRA5 (PV-MXRA5), and they are identical. The amino acid sequences colored in light blue, pink, brown, and gray are derived from exons 3, 4, 5, and 6 of MXRA5. Amino acid sequences colored in purple are derived from exon 7 of MXRA5 and exon 2 of PV-MXRA5, and they are identical. Amino acids in the exon boundary are colored red. AA = amino acids.

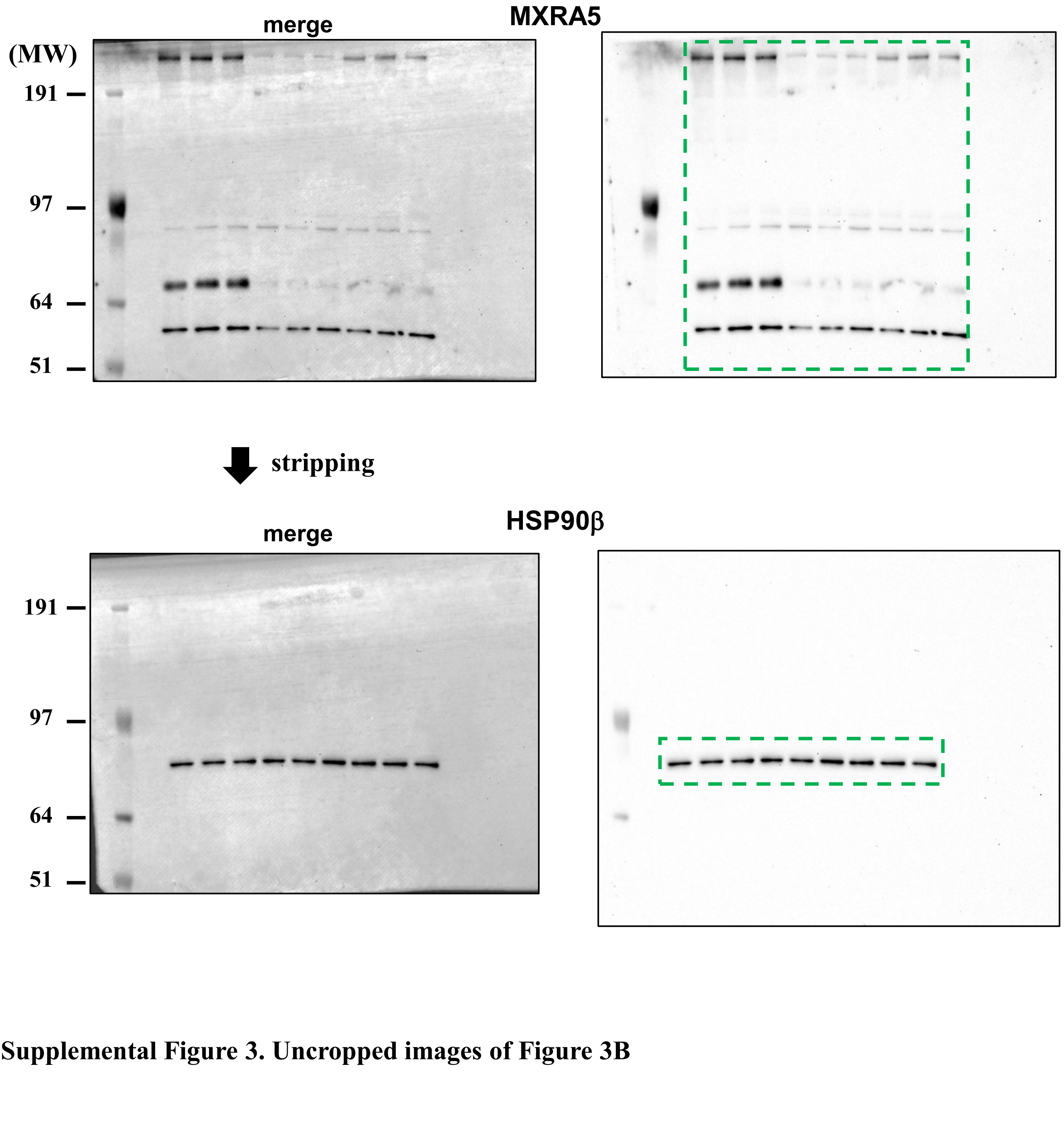


**Supplemental Figure 3. Uncropped images of Figure 3B**

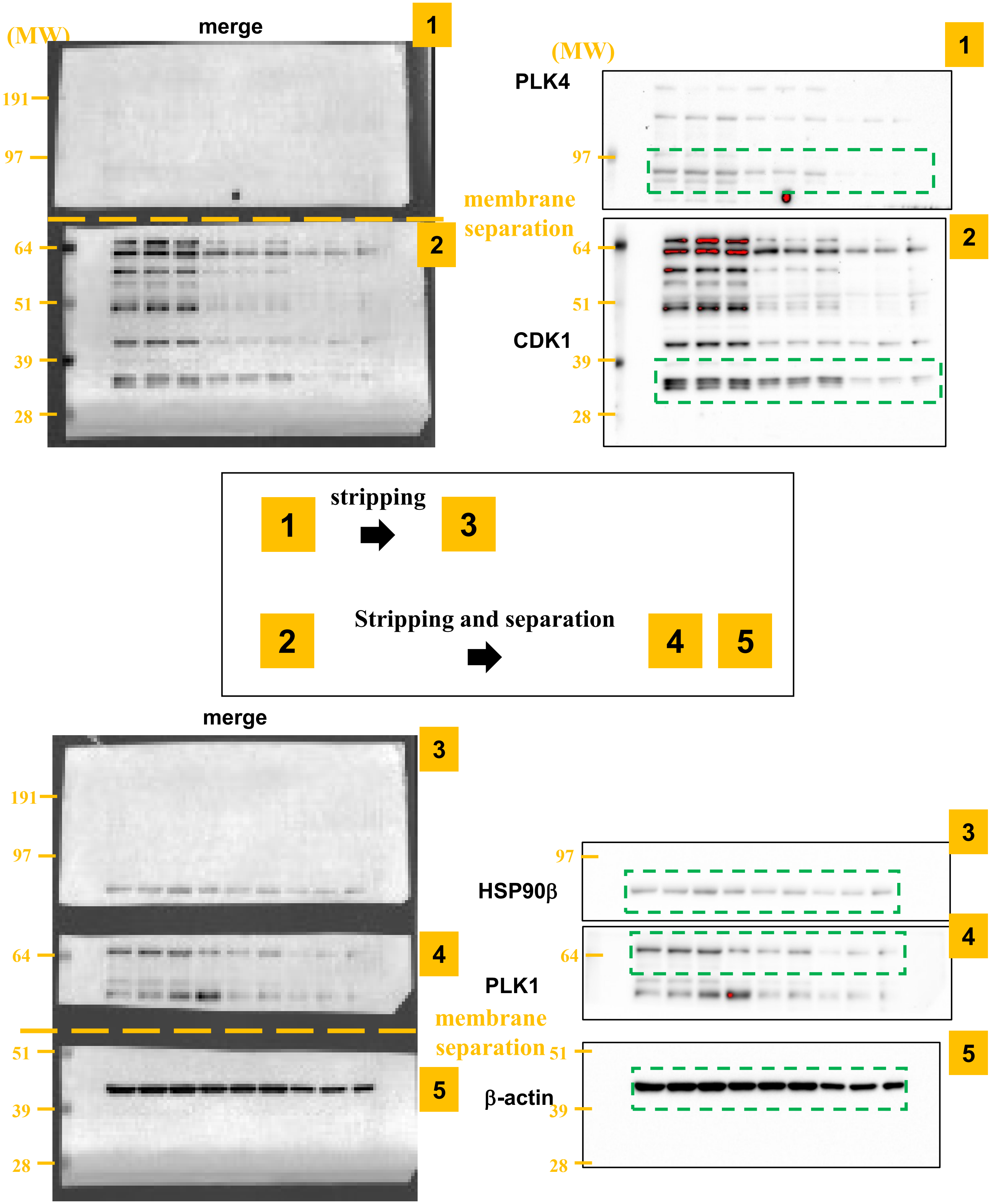


**Supplemental Figure 4. Uncropped images of Figure 4C**

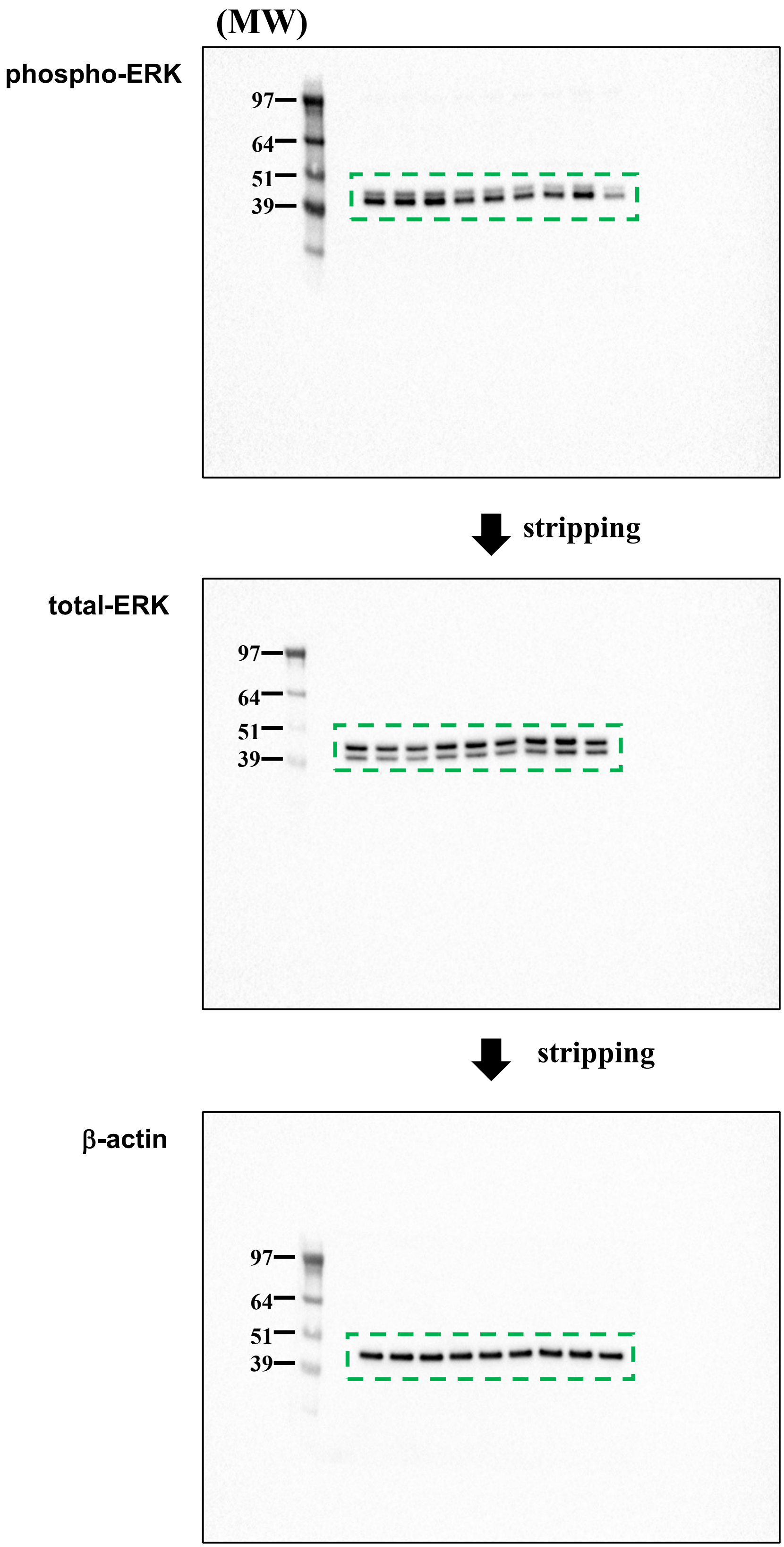


**Supplemental Figure 5. Uncropped images of Figure 4D**

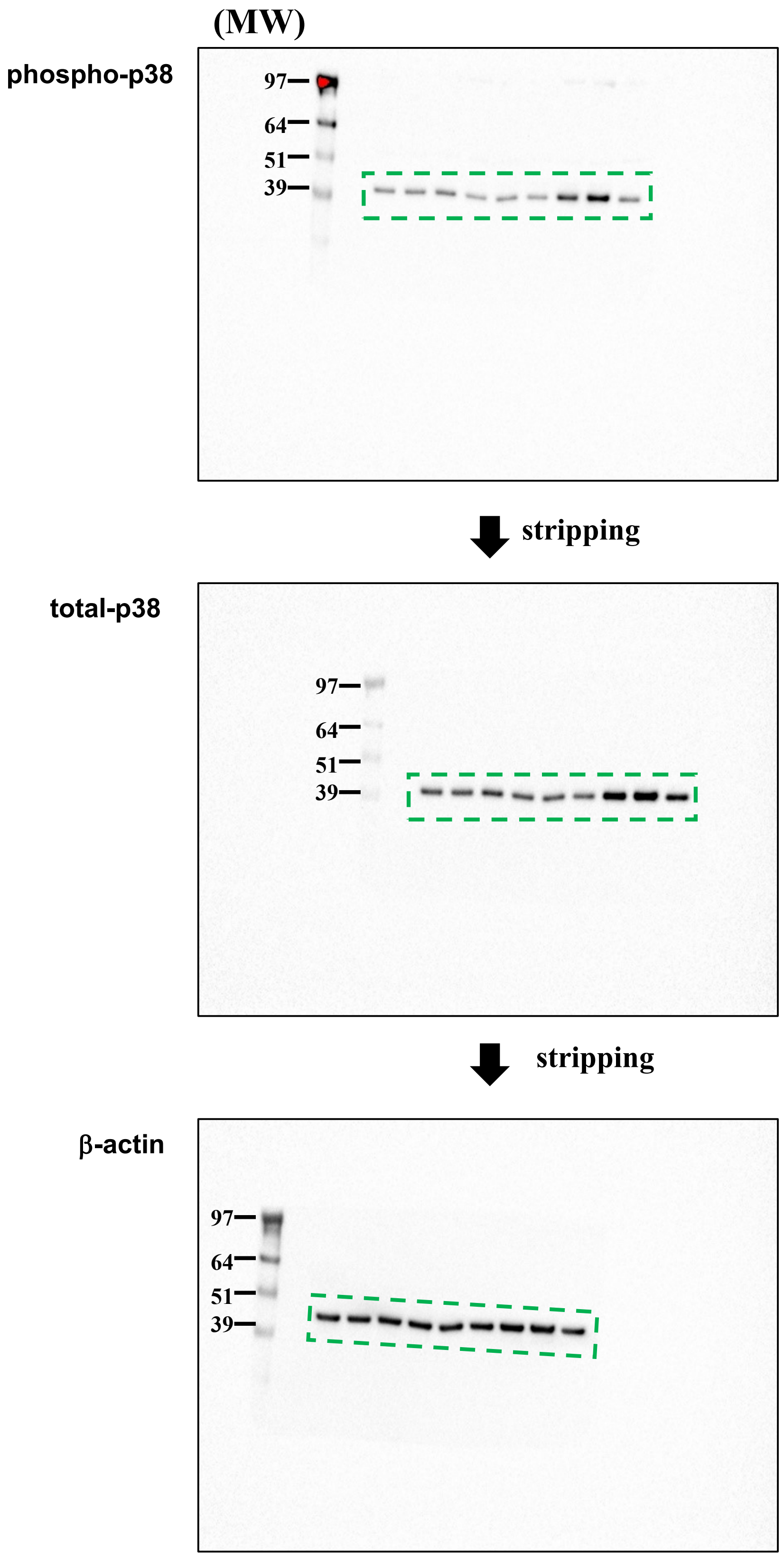


**Supplemental Figure 6. Uncropped images of Figure 4D**

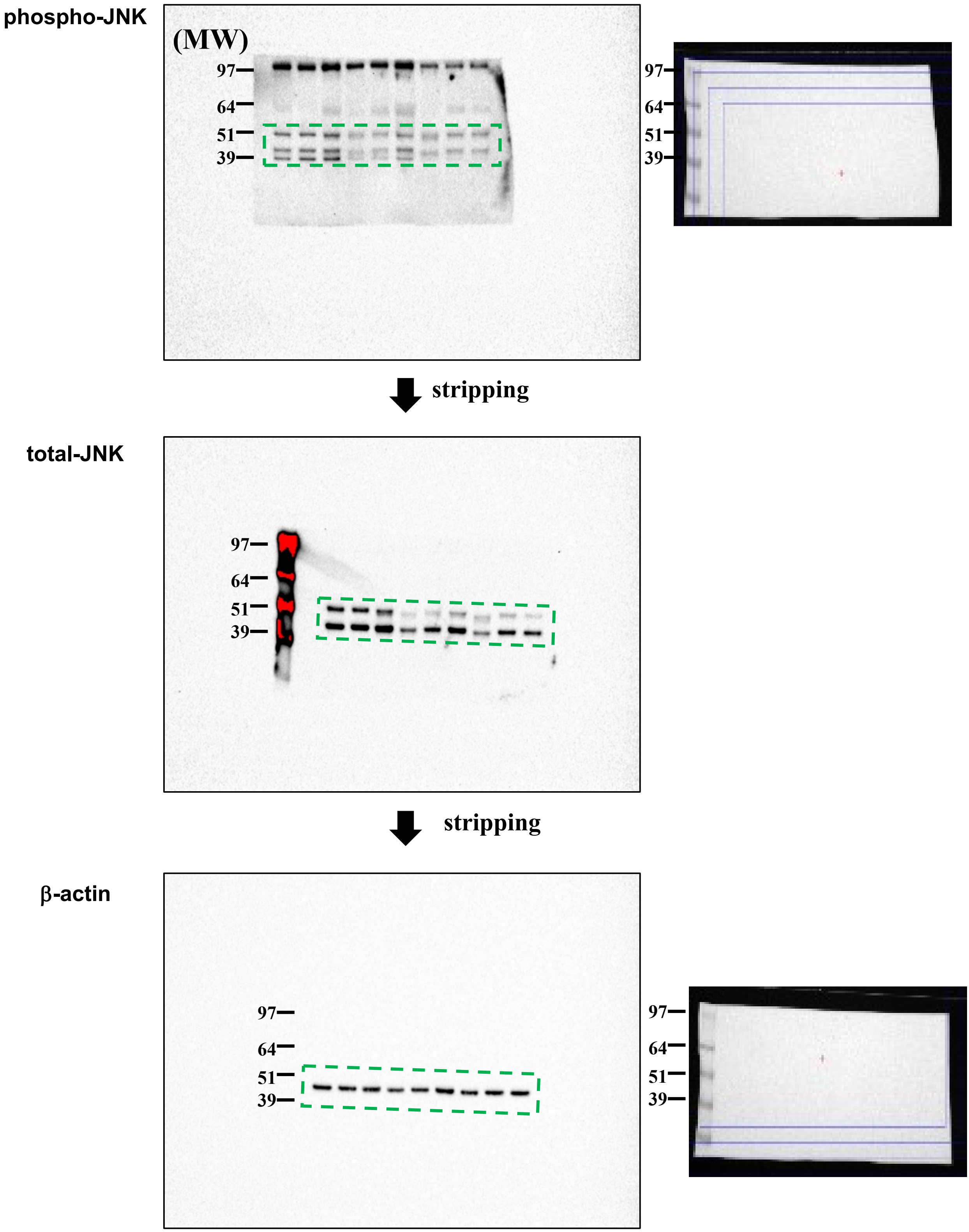


**Supplemental Figure 7. Uncropped images of Figure 4D**

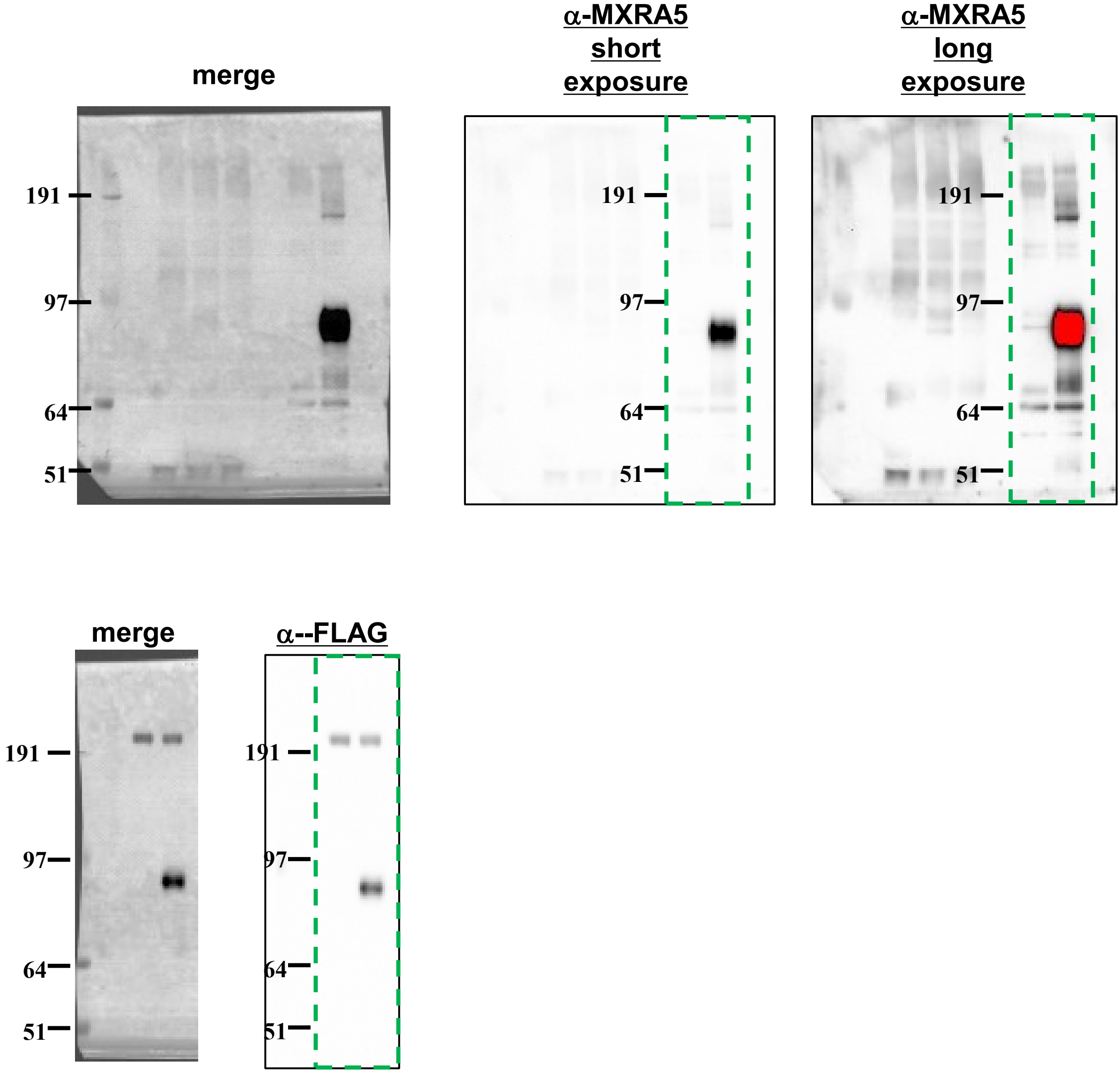


**Supplemental Figure 8. Uncropped images of Figure 5B**
